# Improved Intra-Pixel Sensitivity Characterization Based on Diffusion and Coupling Model for Infrared Focal Plane Array Photodetector

**DOI:** 10.3390/s21248195

**Published:** 2021-12-08

**Authors:** Li Zhong, Xiaoyan Li, Min Zhu, Zhuoyue Hu, Fansheng Chen

**Affiliations:** 1 Key Laboratory of Intelligent Infrared Perception, Chinese Academy of Sciences, Shanghai 200083, China; zhongli@mail.sitp.ac.cn (L.Z.); lixiaoyan@ucas.ac.cn (X.L.); zhumin@mail.sitp.ac.cn (M.Z.); huzhuoyue@mail.sitp.ac.cn (Z.H.); 2 CAS Key Laboratory of Infrared System Detection and Imaging Technology, Shanghai Institute of Technical Physics, Shanghai 200083, China; 3 University of Chinese Academy of Sciences, Beijing 100049, China; 4 Hangzhou Institute for Advanced Study, University of Chinese Academy of Sciences, Hangzhou 310024, China

**Keywords:** intra-pixel sensitivity, infrared focal plane array photodetector, diffusion and coupling model

## Abstract

The high-precision characterization of the intra-pixel sensitivity (IPS) for infrared focal plane array (FPA) photodetector is of great significance to high-precision photometry and astrometry in astronomy, as well as target tracking in under-sampled remote sensing images. The discrete sub-pixel response (DSPR) model and fill factor model have been used for IPS characterization in some studies. However, these models are incomplete and lack the description of physical process of charge diffusion and capacitance coupling, leading to the inaccuracy of IPS characterization. In this paper, we propose an improved IPS characterization method based on the diffusion and coupling physical (DCP) model for infrared FPA photodetector, which considering the processes of generation and collection of the charge, can improve the accuracy of IPS characterization. The IPS model can be obtained by convolving the ideal rectangular response function with the charge diffusion function and the capacitive coupling function. Then, the IPS model is convolved with the beam spot profile to obtain the beam spot scanning response model. Finally, we calculate the parameters of IPS by fitting the beam spot scanning response map with the proposed DCP model based on the Trust-Region-Reflective algorithm. Simulated results show that when using a 3 μm beam spot to scan, the error of IPS characterization based on DCP model is 0.63%, which is better than that of DSPR model’s 3.70%. Experimental results show that the fitting error of the beam spot scan response model based on DCP model is 4.29%, which is better than that of DSPR model’s 8.31%.

## 1. Introduction

Infrared FPA photodetector is widely used in astronomical observation and remote sensing for point target tracking [1,2]. The non-uniformity of the IPS of the infrared FPA photodetector can be ignored when the optical PSF is oversampled. However, when the image is under-sampled, the non-uniformity of IPS has a significant effect on high-precision photometry and astrometry in astronomy as well as point target tracking in remote sensing [3,4,5].

The experimental measurement methods of IPS mainly include the interference pattern method based on frequency domain and beam spot scanning method [6]. Measuring the photodetector based on Michelson interferometer and calculating the IPS based on inverse Fourier transform is an effective method in the frequency domain [7,8]. The optical system of this method is very complex; therefore, the continuously self-imaging grating (CSIG) method has been applied. The CSIG method consists of the sampling of the pixel transfer function by projecting high-resolution periodic patterns without classic optics but using the CSIG illuminated by a plane wave, and the IPS is determined by inverse Fourier transform [9,10]. Although the CSIG method does not require a complex optical system, it can only characterize the average IPS of all pixels in the photodetector array, and the accuracy of IPS characterization for the CSIG method is not high because it cannot consider the physical principle of IPS.

Therefore, the beam spot scanning method is still widely used because it can effectively test the IPS of any pixel. The basic principle of beam spot scanning method is to focus a pixel-sized small beam spot on the pixel and to obtain the pixel response map by beam spot scanning, and then to solve the IPS using certain models and algorithms [6]. The size of IPS profile can be measured by using different sized spots to scan [11], but this method cannot characterize the IPS profile effectively. The fill factor model is also used to fit the beam spot scanning response map of infrared FPA, and the main drawback is that the distribution of IPS cannot be calculated, only the size of the photosensitive area of pixel can be calculated [12]. In some studies, the IPS is constructed by dividing the pixel into a discrete sub-pixel grid or DSPR model, and the pixel response map of beam spot scan is regarded as the convolution of beam spot profile and IPS. Therefore, the Wiener or Lucy–Richardson deconvolution technique are often used to calculate IPS [13,14,15,16]. A disadvantage of deconvolution is that the low-amplitude high-frequency components are suppressed and information on small sensitivity variations in the pixel is lost [17]. Then, the model fitting method can also be used to calculate IPS based on the DSPR model. These include using a Bayesian posterior probability density function to fit the IPS parameter of Euclid Visible Instrument CCD [18] and a forward modeling technique to calculate the IPS of CMOS image sensors [17].

However, these methods based on the DSPR model do not consider the actual physical effects and just calculate the finer grid pixel response, which will result in lower calculation accuracy. Meanwhile, the model fitting method for DSPR model requires a large number of parameters to be calculated with high complexity, which will result in overfitting and inaccurate IPS calculation. When considering the physical principle, the charge diffusion length of HgCdTe infrared FPA photodetector can be calculated based on 1D diffusion equation and the beam spot response map, and the IPS is measured based on the diffusion length [19,20]. In addition, the charge diffusion model and capacitive coupling model are considered for infrared FPA photodetector with hybrid ROIC simultaneously. The magnitude of the charge diffusion and capacitive coupling are then determined by fitting this model to the raw beam spot scanning map data to calculate the IPS [21,22]. However, the beam profile must be known, which needs to be modeled by analytical calculation or tested by the knife-edge scans. Furthermore, more complete infrared FPA response model has been widely used including numerical method [23,24,25], diffusive-probabilistic model [26], and Monte Carlo simulation method [27,28]. However, due to software or algorithm limitations, it is difficult to fit these models with a beam spot scanning map to obtain the appropriate IPS for infrared FPA.

Aiming at the problems of the above methods, we propose an improved physical model based on charge diffusion and capacitive coupling and use the Trust-Region-Reflective algorithm [29] to characterize the IPS of infrared FPA photodetector. Using this improved method, we can still characterize the IPS with high accuracy when the beam profile is unknown or difficult to measure.

## 2. Materials and Methods

### 2.1. Experimental Setup

**Figure 1:**
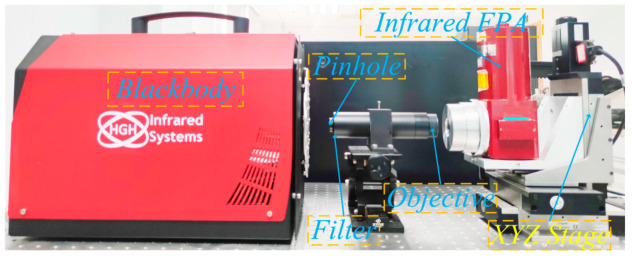
Experimental setup of the intra-pixel sensitivity for infrared focal plane array.

As shown in Figure 1, the experimental setup comprised a high temperature cavity blackbody, pinhole, band pass filter, custom objective lens, high-precision XYZ linear stage, control system, and the data processing system. The specific parameters are shown in Table 1. In addition, the infrared FPA with a cut-off wavelength of 2.5 μm is mounted on an XYZ linear stage.

**Figure 2:**
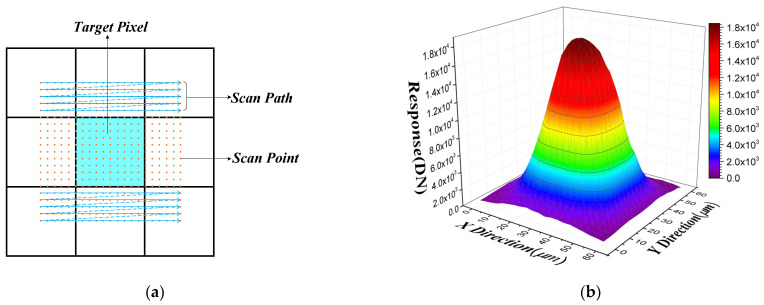
Diagram of the beam spot scan procedure and result: (**a**) schematic diagram of scan procedure; (**b**) beam spot scanning map of infrared FPA photodetector.

In order to achieve the characterization of the IPS for the target pixel, it is necessary to use the small beam spot scanning experimental setup to obtain the response map of the target pixel, and then calculate the parameters of IPS based on the model and algorithm. The experimental steps of spot scanning mainly include focusing and scanning. The beam spot is focused and centered on a target pixel by adjusting the three XYZ linear stages to maximize the signal output from target pixel while also keeping the responses of the surrounding pixels symmetrical. Afterward, the two linear stage perpendicular to the optical axis are adjusted so that the beam spot located at the center of the adjacent pixel in the upper left corner of the target pixel. Finally, this position is used as the starting position of 2D beam spot scanning, which is controlled by the software automatically; the schematic diagram of scan procedure is shown in Figure 2a. The step value of displacement is 3μm, the scanning area is 60 μm × 60 μm, and the number of scanning points is 21 × 21. Because the pitch of the tested infrared FPA photodetector is 30μm, the scanning range can cover the area of the target pixel completely. After completing the experimental scan, the beam spot response map $ScanMap_{i,j}\left(x,y\right)$ of the target pixel $P_{i,j}$ can be obtained, which is shown in Figure 2b.

### 2.2. Improved IPS Model Based on the DCP

Ideally, the IPS of infrared FPA photodetector is uniform, which can be considered as the ideal rectangular window function and expressed as follows:

(1) $$IPS_{i,j;ideal}\left(x,y\right)=rect_{i,j}\left(x,y\right)=\left\{ \begin{array}{l} 1,\cdot \cdot \cdot \cdot \cdot \cdot x,y\in P_{i,j}\\ 0,\cdot \cdot \cdot \cdot \cdot \cdot others \end{array}\right.$$

where $IPS_{i,j;ideal}\left(x,y\right)$ is the IPS of the pixel $P_{i,j}$ in the array.

The basic physical principle for the imaging of HgCdTe infrared FPA photodetector is the photoelectric effect, which converts the incident photons into photoelectrons in the absorber layer; these are then collected by the PN junction. Photoelectrons will undergo a diffusion process in the absorption layer of the infrared FPA photodetector. When the ideal point light source incident is at different positions in the pixel, the probability of photoelectrons diffusing into adjacent pixels is different so the number of collected photoelectrons for target pixel are different, which will cause the non-uniformity of the IPS. The charge-carrier diffusion model can be approximately expressed by a secant model as follows [21]:

(2) $$diff_{i,j}\left(x,y\right)=\sec\;h\left(\frac{\sqrt{{\left(x-x_{ij}\right)}^{2}+{\left(y-y_{ij}\right)}^{2}}}{l_{d}}\right)$$

where $l_{d}$ is the diffusion length of photoelectrons, $\left(x_{ij},y_{ij}\right)$ is the generation position of photoelectrons in the pixel $P_{i,j}$, and the $\sqrt{{\left(x-x_{ij}\right)}^{2}+{\left(y-y_{ij}\right)}^{2}}$ is the distance of the position of charge collected from the position of photoelectrons generated.

A hybrid readout integrated circuit is used for infrared FPA photodetector, where the photoelectrons are collected and transmitted to the readout circuit through an interconnected indium bump. Since the indium bump between adjacent pixels is all metallic, it can be equivalent to a capacitor. When the photoelectrons pass through the indium bump, the capacitive coupling effect will occur, and part of the photoelectrons collected by the target pixel will be coupled to the adjacent pixels. Because the number of coupled photoelectrons is directly related to the number of photoelectrons collected by the target pixel, there will be differences in the coupling signals for the ideal point light source incident at different locations in the pixel, resulting in non-uniformity of IPS. The capacitance coupling model of the infrared FPA photodetector is expressed as follows, which only considers the coupling effect of the nearest four adjacent pixels [30].

(3) $$K_{i,j}=\left(\begin{array}{ccc} 0 & \alpha & 0\\ \alpha & 1-4\alpha & \alpha \\ 0 & \alpha & 0 \end{array}\right)$$

where α is the capacitive coupling coefficient of the infrared FPA photodetector.

Therefore, the IPS model of infrared FPA photodetector can be established based on the above physical process. The actual IPS model is the result of the interaction of ideal rectangle function, charge diffusion, and capacitance coupling model. When only considering the charge diffusion model, the IPS is the convolution of the response of the ideal rectangular window function and the charge diffusion model function, which is expressed as follows:

(4) $$IPS_{i,j;diff}\left(x,y\right)=rect_{i,j}\left(x,y\right)*diff_{i,j}\left(x,y\right)$$

where * denotes the convolution operator.

When considering the capacitive coupling model, the output signal of the target pixel is the signal collected in the target pixel plus the signal coupled from the nearest four adjacent pixels, minus the signal coupled from the target pixel into the adjacent pixel. After considering charge diffusion and capacitive coupling model simultaneously, the IPS can be expressed as follows,

(5) $$\begin{array}{l} IPS_{i,j}\left(x,y\right)=IPS_{i,j;diff}\left(x,y\right)-4\alpha \cdot IPS_{i,j;diff}\left(x,y\right)+\\ \left(IPS_{i-1,j;diff}\left(x,y\right)+IPS_{i+1,j;diff}\left(x,y\right)+IPS_{i,j-1;diff}\left(x,y\right)+IPS_{i,j+1;diff}\left(x,y\right)\right)\cdot \alpha \end{array}$$

(6) $$IPS_{i,j}\left(x,y\right)=\sum_{m=i-1}^{i+1}\sum_{n=j-1}^{j+1}IPS_{m,n;diff}\left(x,y\right)\cdot K_{i,j}\left(m+2-i,n+2-j\right)$$

and the Equation (5) can be simplified to the Equation (6) by using the coupling kernel $K_{i,j}$.

### 2.3. Parameters Solving of IPS Based on the DCP

When the beam spot scanning response map and IPS model are obtained, the next step is to solve the parameters of IPS. Since the objective lens of the experimental setup works in focusing mode, the profile of the beam spot on the target pixel can be approximately expressed by a two-dimensional Gaussian function model. In addition, due to the error of linear stage, the actual position of scanning is different from the expected position of scanning. Therefore, the deviation parameter in *x* and *y* scanning direction can be introduced into the beam spot distribution function and expressed as follows:

(7) $$G_{i,j}\left(x,y\right)=A\cdot \exp\left(-\frac{{\left(x-x_{ij}-\Delta x\right)}^{2}+{\left(y-y_{ij}-\Delta y\right)}^{2}}{2\sigma^{2}}\right)$$

where A is the intensity of the beam spot and σ is the size of the beam spot, Δx and Δy are the deviation parameter in *x* and *y* scanning direction.

Therefore, the theoretical model expression of the beam spot scanning response map of the target pixel can be obtained and expressed as follows, which is the convolution of the beam spot distribution function and the IPS of the target pixel $P_{i,j}$.

(8) $$M_{i,j}\left(x,y;paras\right)=IPS_{i,j}\left(x,y\right)*G_{i,j}\left(x,y\right)$$

where paras is the model parameters $\left[A,\sigma ,l_{d},\alpha ,\Delta x,\Delta y\right]$, including the intensity and size of beam spot, charge diffusion length, capacitance coupling coefficient, as well as the parameters of deviation in *x* and *y* scanning direction.

Then, we extract the IPS by fitting the beam spot scanning response map with the proposed model expression given by Equation (8), using a goodness-of-fit measure as follows:

(9) $$para=\underset{para}{argmin}{\sum_{k=1}^{N}\left[ScanMap_{i,j}\left(x_{k},y_{k}\right)-M_{i,j}\left(x_{k},y_{k};para\right)\right]}^{2}$$

where *N* is the number of scanning points of beam spot—we only use the scanning points in the target pixel because of higher SNR for these points—and $x_{k},y_{k}$ are the coordinates of the *k*-th scanning point. For the nonlinear least-squares fitting procedure, we use the Trust-Region-Reflective algorithm to optimize the parameters. This algorithm is a subspace trust-region method based on the interior-reflective Newton method, and each iteration involves the approximate solution of a large linear system using the method of preconditioned conjugate gradients. The solver’s stopping criteria include function tolerance, step tolerance, and maximum number of iterations allowed. We set the function tolerance and step tolerance $10^{-6}$, and the maximum iterations is 500.

## 3. Results and Discussion

### 3.1. Evaluation Criteria

Some evaluation criteria are determined to verify the performance of the improved model for the IPS characterization of the infrared FPA photodetector. The error between the calculated IPS and the true IPS can be used to evaluate the performance of model directly, which is expressed as follows:

(10) $$IpsCalError=\frac{1}{m\times n}\sum_{m=1}^{nums}\sum_{n=1}^{nums}\left|\frac{IPS_{i,j;cal}\left(m,n\right)-IPS_{i,j;true}\left(m,n\right)}{IPS_{i,j;true}\left(m,n\right)}\right|$$

where (m,n) is the index of the subpixel, nums is the number of subpixel, $IPS_{i,j;cal}\left(m,n\right)$ is the calculated IPS of pixel $P_{i,j}$, $IPS_{i,j;true}\left(m,n\right)$ is the true IPS of pixel $P_{i,j}$.

However, as the true value of the IPS cannot be determined for the experimental test, the error of fitting for experimental beam spot scanning response map with the model in Equation (8) is used to evaluate the performance of the model. The best-fit parameters of IPS model are $para^{\prime }=\left[A^{\prime },\sigma^{\prime },l_{d}^{\prime },\alpha^{\prime },\Delta x^{\prime },\Delta y^{\prime }\right]$. The fitting error is defined as the mean difference between the experimental scanning response map and model fitting value for all scan position, which can be expressed as follows:

(11) $$FittingError=\frac{1}{N}\sum_{k}^{N}\left|\frac{ScanMap_{i,j}\left(x_{k},y_{k}\right)-M_{i,j}\left(x_{k},y_{k};para^{\prime }\right)}{ScanMap_{i,j}\left(x_{k},y_{k}\right)}\right|$$

### 3.2. Simulation Results

**Figure 3:**
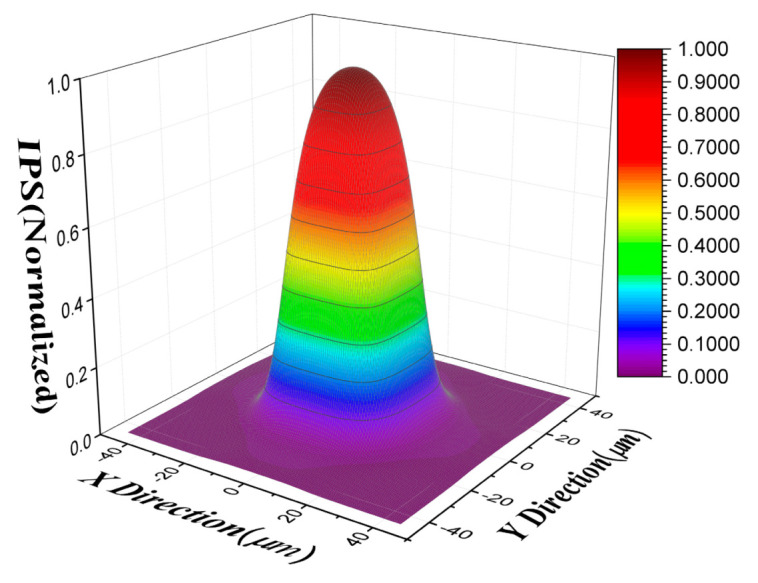
True IPS of the simulation.

We first validated the proposed model and algorithm in simulation data. The charge diffusion length, capacitance coupling coefficient, and the pixel pitch were set as 3 μm, 1%, and 30 μm, respectively. The true IPS of the simulation is shown in Figure 3. Due to the inter-pixel crosstalk introduced by the charge diffusion and capacitance coupling, the target pixel has a response value when point light source illuminates the adjacent pixels. Therefore, the simulated IPS of the target pixel is distributed over the area of 3 × 3 pixels and the response in other area is ignored.

**Figure 4:**
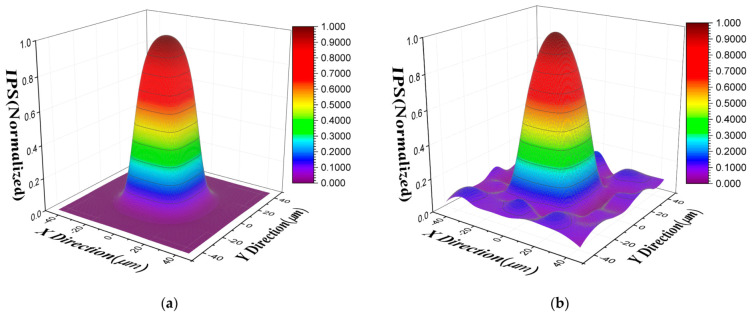
Calculated IPS for simulated data: (**a**) calculated IPS based on the DCP model; (**b**) calculated IPS based on the DSPR model.

Then we simulated the beam spot to scan the target pixel and obtained the simulated scanning response map, and the size of the simulated beam spot was 3 μm. The IPS is then solved based on the proposed DCP model and DSPR model, which can be shown in Figure 4. It can be noticed that the calculated IPS based on the DCP model is closer to the true simulated IPS than the DSPR model.

**Figure 5:**
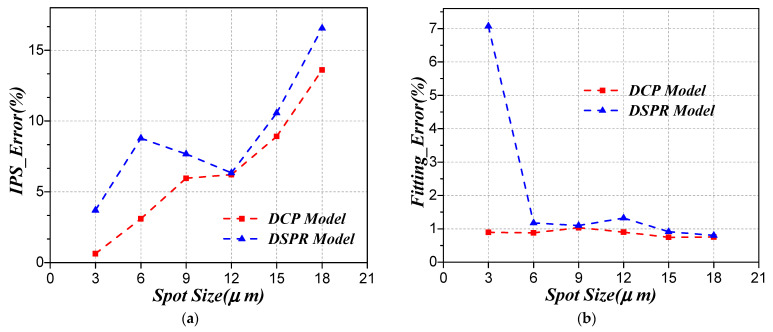
IPS calculated error and fitting error of simulated data for different models: (**a**) IPS calculated error versus the size of simulated beam spot; (**b**) fitting error versus the size of simulated beam spot.

In order to further quantitatively compare the performance of different models of IPS characterization, we simulated different sizes of beam spot to scan the target pixel and calculate the IPS based on different models. The IPS calculated error and fitting error versus different size of simulated beam spot are shown in Figure 5 and listed in Table 2. It can be noticed that the IPS calculated error and fitting error based on the DCP model is smaller than the DSPR model for all different sizes of simulated beam spot. In addition, the calculation error of IPS based on DCP increases as the spot size increases, and the fitting error based on these two models is not very relevant to the size of the beam spot. From the above analysis, the proposed improved DCP model is better than the DSPR model for IPS characterization, and we can use smaller beam spot to scan the target pixel in order to obtain better IPS calculated results.

### 3.3. Experimental Results

**Figure 6:**
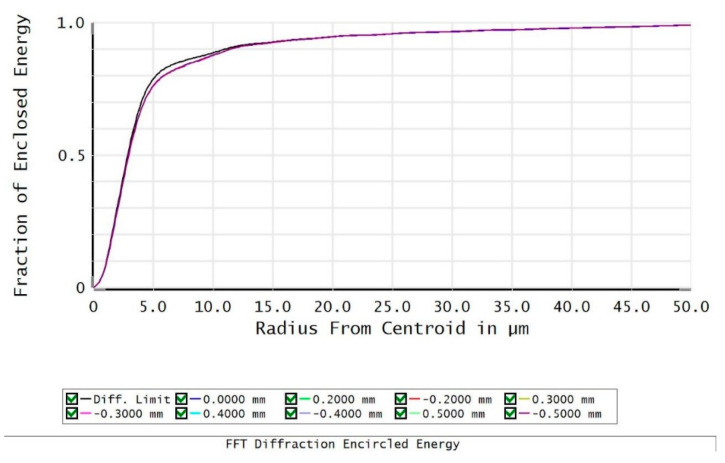
Fraction of enclosed energy of optical system using ZEMAX simulation.

We then validated the proposed model and algorithm in experimental data. The target pixel in the short-wave infrared FPA photodetector was scanned by the experimental setup, which is described in chapter 2.1. By fitting the scanning response map with the DCP model, we can obtain the parameters of the photodetector and beam spot. The calculated parameters include charge diffusion length, capacitance coupling index, and size of the beam spot, which are 2.908 μm, 0.837%, and 4.370 μm, respectively. In order to verify the reliability of the results, we used optical simulation software ZEMAX to simulate the optical system and calculate the relationship between the fraction of enclosed energy and the radius, which is shown in Figure 6. It can be seen from the simulation results that when the fraction of enclosed energy is 68%, the corresponding radius is about 3 μm. The beam spot size obtained through experiments and models is 2.908 μm, which is the standard deviation of the Gaussian distribution function, and the corresponding fraction of enclosed energy is also 68%. Therefore, the consistency of the experimental measurement and theoretical simulation of the beam spot size proves the reliability of our results.

**Figure 7:**
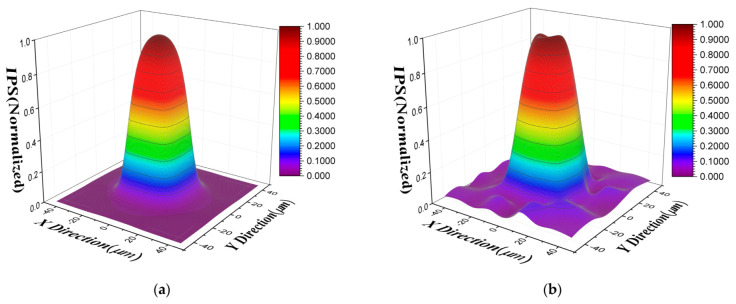
Calculated IPS of target pixel in the short-wave infrared FPA photodetector: (**a**) calculated IPS based on the DCP model; (**b**) calculated IPS based on the DSPR model.

By substituting these calculated parameters into the IPS model in Equation (6), the IPS of the target pixel in the short-wave infrared FPA photodetector was calculated and is shown in Figure 7a. It can be seen that the IPS of the target pixel has a response value even at the position of the adjacent pixel due to the charge lateral diffusion and capacitance coupling, which is consistent with the simulated results.

**Figure 8:**
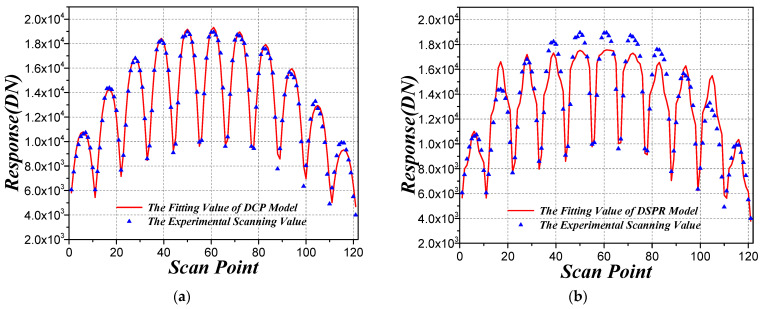
Beam spot scanning response map of the experiment and the fitting value of model (**a**) based on the DCP model; (**b**) based on the DSPR model.

In order to evaluate the performance of the DCP model for the IPS characterization of the experimental data, the beam spot scanning response map of experiment and the fitting value of DCP model in target pixel are plotted in Figure 8a. The fitting error of the DCP model for the target pixel is 4.29%.

Then, in order to compare the proposed improved DCP model with the DSPR model, we also calculated the IPS of the target pixel based on the DSPR model, which is shown in Figure 7b. It can be seen that there exist some fluctuations for the calculated IPS response in adjacent pixels based on the DSPR model, which is mainly caused by the noise of the experimental measurement. Therefore, we found that the DCP model is less affected by noise than the DSPR model. The beam spot scanning response fitting value of DSPR model was also calculated, which is shown in Figure 8b. It can be seen that the fitting value of DCP model is closer to the experimental scanning value than the fitting value of DSPR, and the fitting error of DSPR model for the target pixel is 8.31%, which is higher than the fitting error of DCP model. From the above analysis, the proposed improved DCP model is more accurate than the DSPR model for IPS characterization in experimental data.

In addition, Figure 7a shows that the calculated IPS using DCP does not show any significant response in adjacent pixels, while the calculated IPS using DSPR model shown in Figure 7b has a certain response in adjacent pixels. This is mainly for two reasons. First, the parameters to be fitted are the response values of each discrete sub-pixel point in 3 × 3 pixels for DSPR model, and the IPS response of adjacent pixels has a low contribution to the central pixel and a low signal-to-noise ratio. The influence of noise is directly reflected on the IPS, so it can be found that the IPS on the outer edge of the adjacent pixels has a certain response and fluctuation. However, the parameters to be fitted are only charge diffusion length and capacitive coupling coefficient for the DCP model, and noise mainly affects the accuracy of these two parameters. The calculated IPS does not directly reflect the influence of noise, which is only related to the value of calculated diffusion length and capacitive coupling. When these two values are small, the IPS response is almost zero at the edge of adjacent pixels far from the central target pixel. Second, the algorithm will also affect the results of the DSPR model. The convergence and robustness of the optimal algorithm will be poor due to the large number of parameters to be fitted. Therefore, the IPS response in adjacent pixels is more likely to be affected by the performance degradation of the optimal algorithm due to its relatively small contribution to the weight of objective function.

## 4. Conclusions

In this paper, a high-precision characterization method of the IPS for the infrared FPA photodetector based on the DCP model was proposed, which considers the physical process of charge diffusion and capacitive coupling. With the simulated and experimental beam spot scanning response map, we calculated the IPS based on the improved DCP model and the traditional DSPR model. From the results and analysis, it can be seen that the improved DCP model is more accurate than the traditional DSPR model for IPS characterization, and we can calculate the IPS without knowing the profile of the beam spot, which is beneficial for the high-precision measurement of under-sampled infrared point targets.

## Figures and Tables

**Table 1:** Specifications of experimental setup.

Equipment	Parameters	Manufacturer
Blackbody	Temperature range: 50~1250 °C Temperature accuracy: 0.1 °C The emissivity: >0.99	HGH
Pinhole	Diameter: 5 μm	Custom
Filter	Wavelength: 2–2.5 μm	Thorlabs
Object Lens	F#: 2 Focal length: 40 mm	Custom
XYZ Linear Stage	Minimum increment: 0.3 μm Uni-directional repeatability: ±0.2 μm Bi-directional repeatability: ±2.2 μm	Newport

**Table 2:** IPS calculated error and fitting error of different size of simulated beam spot.

Spot Size (μm)	IPS Error (%)		Fitting Error (%)	
	DCP	DSPR	DCP	DSPR
3	0.63	3.70	0.90	7.07
6	3.10	8.78	0.88	1.18
9	5.96	7.67	1.03	1.10
12	6.21	6.34	0.90	1.32
15	8.92	10.56	0.74	0.91
18	13.60	16.56	0.76	0.80

## Data Availability

Data sharing not applicable.
